# A prospective cohort study of stroke mortality and arsenic in drinking water in Bangladeshi adults

**DOI:** 10.1186/1471-2458-14-174

**Published:** 2014-02-18

**Authors:** Mahfuzar Rahman, Nazmul Sohel, Mohammad Yunus, Mahbub Elahi Chowdhury, Samar Kumar Hore, Khalequ Zaman, Abbas Bhuiya, Peter Kim Streatfield

**Affiliations:** 1 ICDDRB, 68 Shahid Tajuddin Ahmed Sarani, Mohakhali, Dhaka 1212, Bangladesh; 2 University of Chicago Research Bangladesh, House 338, Road 24, New DOHS, Mohakhali, Dhaka 1212, Bangladesh; 3 Department of Clinical Epidemiology and Biostatistics, McMaster University, 1280 Main Street West, Hamilton ON L8S 4K1, Ontario, Canada

## Abstract

**Background:**

Arsenic in drinking water causes increased coronary artery disease (CAD) and death from CAD, but its association with stroke is not known.

**Methods:**

Prospective cohort study with arsenic exposure measured in well water at baseline. 61074 men and women aged 18 years or older on January 2003 were enrolled in 2003. The cohort was actively followed for an average of 7 years (421,754 person-years) through December 2010. Based on arsenic concentration the population was categorized in three groups and stroke mortality HR was compared to the referent. The risk of stroke mortality Hazard Ratio (HR) and 95% Confidence Interval was calculated in relation to arsenic exposure was estimated by Cox proportional hazard models with adjustment for potential confounders.

**Results:**

A total of 1033 people died from stroke during the follow-up period, accounting for 23% of the total deaths. Multivariable adjusted HRs (95% confidence interval) for stroke for well water arsenic concentrations <10, 10-49, and ≥50 μg/L were 1.0 (reference), 1.20 (0.92 to 1.57), and 1.35 (1.04 to 1.75) respectively (P_trend_=0.00058). For men, multivariable adjusted HRs (95%) for well water arsenic concentrations <10, 10-49, and ≥50 μg/L were 1.0 (reference), 1.12 (0.78 to 1.60), and 1.07 (0.75 to 1.51) respectively (P_trend_=0.45) and for women 1.0 (reference),1.31 (0.87 to 1.98), and 1.72 (1.15 to 2.57) respectively (P_trend_=0.00004).

**Conclusion:**

The result suggests that arsenic exposure was associated with increased stroke mortality risk in this population, and was more significant in women compared to men.

## Background

An estimated 100 million people worldwide are currently exposed to elevated concentrations of arsenic in their drinking water [1]. Internal cancers (lung, bladder, kidney and liver) and numerous non-cancer diseases are associated with elevated levels of arsenic in drinking water [2]. Arsenic contamination in drinking water is an emerging public health issue globally, particularly in Bangladesh, where the problem has been called the “greatest mass poisoning in history” [3].

Many studies supports the fact that arsenic in drinking water causes increased coronary artery disease (CAD) and death from CAD, but the evidence concerning cerebrovascular disease (stroke mortality) is not conclusive. Stroke mortality is one of the causes of death, is responsible for 10% of deaths globally [4]. Only a few studies have examined the impact of arsenic exposure on stroke [5-7]. Environmental arsenic exposure causes excess stroke mortality among black foot disease (BFD) patients and residents of Taiwan [5-7]. BFD means peripheral atherosclerosis resulting in dry gangrene and spontaneous amputation of affected extremities and the disease was named for its most striking clinical feature—blackish discoloration of the feet or hands. Occupational arsenic exposure has also been implicated to stroke mortality [8]. However, most population based studies were either ecological or occupational cohort studies. Ecologic correlation studies may have the problem of ecological fallacy, and occupational cohorts may have the limitations of multiple exposures. Thus, only limited conclusions can be drawn from these studies.

Given that a high proportion of stroke events result in death, even a small increase in risk associated with arsenic exposure can mean a large number of excess deaths in the exposed population. In our earlier study, we reported increased risk of adult cardiovascular mortality in Bangladesh associated with arsenic exposure [9]. However, a limitation of that study was that it lacked individual exposure data and used household level exposure for the subjects. The Arsenic exposure and health consequences in Matlab (As Mat) study [10] includes participants exposed to a wide range of arsenic concentrations in drinking water at baseline (0.5 to 3644 μg/L), using individual level exposure provides a unique opportunity to investigate the cerebrovascular effects of exposure to arsenic at low-to-moderate concentrations. By following up this existing cohort we assess the risk of stroke mortality overall as well as by gender and subtype of stroke.

## Methods

### Study area and design

Matlab is a typical rural area of Bangladesh located 55 kilometres southeast of the capital, Dhaka. Matlab is an area severely affected by arsenic contamination of the drinking water, with numerous established adverse health consequences [9,10]. All individuals gave written informed consent to participate. An icddrb (International Centre for Diarrhoeal disease Research Bangladesh) institutional review committee and the icddrb Ethical Review Committee approved the baseline study. A mitigation program was initiated in collaboration with Bangladesh Rural Advancement Committee (BRAC), Bangladesh.

**Figure 1:**
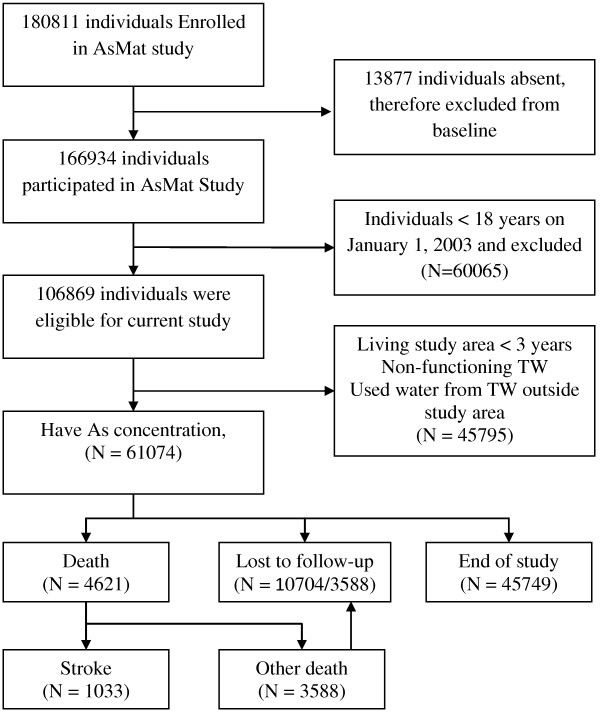
**Study flow chart.** The cohort comprised all inhabitants in Matlab, Bangladesh, age≥18 years on January 1, 2003, living in the study area for at least 3 years (reducing loss-to-follow-up); and primary users of the tested well, with follow-up to death or December 31, 2010 (closed cohort).

We assembled our present study cohort, the Arsenic Adult Cohort (AAC) from our baseline study which consisted of screening and obtaining the lifetime drinking water history of 166,934 individuals (74,408 male, 92,526 female). Detail of study location describe elsewhere [11]. From population database 220, 000, our primary screening in 2002-2003 included all individuals greater than 4 years of age were included. Individuals were excluded who were not living and drinking water of a source in Matlab at least once per week. A total of 180, 811 individuals were eligible (Figure 1) and visited their homes during the study period (January 2002-August 2003). Thus, 166, 934 individuals (92% of eligible) were interviewed and examined. For this cohort, we recruited 61,074 adults who met our eligibility criteria: age≥18 years on January 1, 2003, living in the study area for at least 3 years (reducing loss-to-follow-up); and primary users of the tested well (n=13,286). Participants were followed from January 01, 2003 until December 31, 2010. Information on demographic and socio-economic information was collected at baseline. Community health research workers visit every household on a monthly basis to update information on demographic events, i.e. marriages, births, deaths, in- and out-migrations, as well as to collect information on morbidity of children below 5 years of age and of women of childbearing age. Socio-economic information is also recorded by periodic censuses.

Demographic information were collected monthly basis and socioeconomic information collected for every 5 years. We used SES for 2005. This study used prospectively collected health and demographic data collected monthly through the “Health and Demographic Surveillance System (HDSS)” during the period from January 2003 to December 2010 collected through verbal autopsy (VA) [12]. Verbal autopsy procedures are widely used for estimating cause specific mortality in areas where there is no medical record or formal medical attention given.

Follow-up time in person-years was calculated as the number of days between the baseline interview to date of death, out migration date or the end of the study period (December 31, 2010), whichever came first. Participants with an accident-related cause of death such as road traffic accident, drowning or other accidental deaths or died from a cause other than stroke or alive were censored.

### Stroke mortality data

Our outcome of interest was death (18 years and above) from stroke (International classification of diseases, 10^th^ revision codes, I61-I69) between January 1, 2003 and December 31, 2010. Causes of deaths including stroke were recorded based on routine VA conducted by specially trained field staff of HDSS who were unaware of the well water arsenic concentration. After appropriate training, these field staff interviewed close relatives of the deceased using a structured verbal-autopsy questionnaire that captures signs and symptoms of diseases/conditions that were present prior to death and medical consultations that occurred before death. Later, two physicians independently assigned the underlying causes of deaths. If there was a disagreement, a third physician resolved cause of deaths [12]. They coded the causes by use of the World Health Organization (WHO) tenth revision of the International Classification of Diseases (ICD-10). Assignment of causes of death was done in accordance with the verbal autopsy standards that have been developed by the INDEPTH network and the WHO [12]. VA is a well-known instrument for ascertaining the cause of death based on information obtained from close relatives through systematic retrospective questioning [12]. Stroke deaths included: Intracerebral hemorrhage, ICD code I61=2; other non-traumatic hemorrhage, ICD code I62=1; cerebral infarction, ICD code I63=9; Stroke, ICD code I64=480; ICD code I67=6; other cerebrovascular diseases, and Sequelae of cerebrovascular disease, ICD code I69=535 deaths.

### Arsenic exposure assessment

At baseline, 13, 286 tube-wells were functional and tested for arsenic content. The field teams interviewed all individuals regarding their water-consumption history, and recorded the water sources used, including location, during each calendar year. Water samples were collected in two 20 ml polyethylene vials. The vials contained acid to prevent precipitation. They were kept at –20°C until analysis. The concentrations of arsenic were determined in duplicate by hydride generation atomic absorption spectrophotometer (HG-AAS, Shimadzu Model AA-6800) at Dhaka icddr,b laboratory. The limit of detection (LOD) was 1 μg/L. In the calculations, results below LOD were assigned a value of 0.5 μg/L. Analytical performance was controlled by analysis of standard reference material, internal water quality control samples, and inter-laboratory comparison (Karolinska Institutet). Details of study methodology and results are given elsewhere [10].

### Other study variables

Other covariates were derived from HDSS data. In our earlier study, increased risks for death were observed with socioeconomic conditions and education [9]. Asset scores were used based on a household level socioeconomic census in 2005 that contained household-level information. Using available information for each household a score was then generated through principal component analysis. SES scores were first developed for each household in the study sample. To create the categorical variable, SES scores were sorted from lowest to highest and divided into equal quintile; higher quartiles reflect higher SES. Ultimately, we chose to include SES as categorical variable. All the scores were categorized into five groups ranging from the lowest (poorest) to highest (richest). Those with missing SES information (N=867) were imputed by the series mean. A sensitivity analysis was used to examine if the relationship differed when imputed SES was included in the analysis. Our sensitivity analysis revealed that the outcome remained stable after including the imputed value of missing SES. Other socioeconomic and demographic factors included age at baseline (years), sex and years of education.

### Statistical analysis

Baseline age, gender, asset score, education and baseline arsenic exposure were compared between baseline cohort member and deaths, and between participating and loss to- follow participants, supported by Chi square testing. The mortality risks in relation to arsenic exposure were estimated by Cox proportional hazards models, adjusting for potential confounders, and adjusted Hazard Ratios (HRs) were calculated along with 95% confidence intervals (95% CI). First we assessed crude association and then adjusted for covariates. A covariate was identified as a potential confounder if associated with exposure and outcome at p≤0.10 significance level. Potential confounders that were found to change the effect estimates by 5% or more were included in the adjusted multivariate models. Baseline exposure was divided into three groups (<10, 10-49, and ≥50 μg/L) with the lowest arsenic exposure level used as reference. Adjusting for sex, SES, education, and baseline age, we plotted cumulative hazard functions for each exposure category. All analyses were done using SPSS 15·0 (SPSS Inc, Chicago, Illinois) software.

## Results

A total of 61 074 participants met the eligibility criteria and were included in the cohort. We observed 4 429 total deaths, excluding accidental deaths (n=192), from an observation of 421 754 person-years. There were 1033 stroke deaths (ICD codes I61 to I69) accounting for 23% of total mortality and yielding a mortality rate of 244.9 per 100 000 person-years. 10,704 (15.5%) individuals were lost to follow-up during the study period, however relatives reported being alive. The majority of those lost to follow up moved-out due to a new job, marriage, or following children. The mean (±SD) age at baseline was 37·0±16·3 years and there were no age difference between gender (men vs. women 37·2±17·2 *vs.* 36·9±16·3 years). Baseline water samples ranged from 0.5 to 3 644 μg/L and the median well water arsenic concentration at baseline was 86.8 μg/L (IQR [inter-quartile range], 20.5-262.0) for individuals who survived (n=58,179) and 101.3 μg/L (IQR 20.5-263.4 μg/L) for those who died.

Table 1 shows the distribution of demographic and exposure characteristics for the baseline cohort and for those who died from stroke. The crude overall rates for death were higher among men than women (291.5 per 100 000 person-years *vs.* 212.6 per 100 000 person-years, P<0.01). There was difference between age distribution, sex, education categories by deceased and survivors (P<0.001). The crude death rate was more than three times higher in the “no education” group compared with participants with secondary education level. The crude death rate was more than double in the “no education” group compared with participants with primary education level.

**Table 1:** Selected characteristics of participants in relation to vital status, age at baseline, sex, education year, exposure and SES

**Variables**	**Baseline arsenic in well water**		**Baseline cohort, N=61,074**		**Stroke deaths, N=1,033**		**Crude death rate per 1,000 person-years**	**P value**
	**Mean**	**SD**	**n**	**%**	**N**	**%**		
Age								
18-30	164.2	182.4	18,927	31.0%	5	.5%	0.04	<0.0001
31-60	161.9	180.4	32,963	54.0%	187	18.1%	0.76	
+60	162.9	178.9	9,184	15.0%	841	81.4%	14.41	
Sex								
Male	162.1	179.5	25,967	42.5%	503	48.7%	2.92	<0.001
Female	163.3	181.8	35,107	57.5%	530	51.3%	2.13	
Education								
0	169.8	181.6	23,771	38.9%	639	61.9%	3.7	<0.001
1-5	167.5	183.5	18,810	30.8%	272	26.3%	1.1	
+6	149.0	176.3	18,493	30.3%	122	11.8%	0.7	
SES								
1	183.2	186.1	9,039	14.8%	142	13.7%	2.2	<0.50
2	170.3	181.1	10,741	17.6%	188	18.2%	2.5	
3	167.9	183.6	12,687	20.8%	232	22.5%	2.7	
4	164.5	178.2	13,445	22.0%	213	20.6%	2.3	
5	139.6	174.8	15,162	24.8%	258	25.0%	2.5	
Baseline arsenic exposure								
<10	1.7	2.0	5,566	9.1%	62	6.0%	1.6	<0.05
10-49	21.1	7.2	22,686	37.1%	375	36.3%	2.4	
+50	102.2	30.5	32,822	53.7%	596	57.7%	2.6	

Table 2 shows no significant difference in exposure distribution between included or excluded persons of this cohort. There are differences in age, sex, and education levels.

**Table 2:** Comparison of the distribution of age, sex, years of education, SES characteristics, and baseline exposure for the total cohort and those lost to follow up

**Characteristics**	**Variables**	**Participated**		**Loss to follow-up**		**P value**
		**N**	**%**	**N**	**%**	
Age						
	18-30	11911	23.6%	7016	65.5%	<0.001
	31-60	29775	59.1%	3188	29.8%	
	+60	8684	17.2%	500	4.7%	
Sex						
	Male	20482	40.7%	5485	51.2%	<0.001
	Female	29888	59.3%	5219	48.8%	
Education						
	0	21684	43.0%	2087	19.5%	<0.001
	1-5	15809	31.4%	3001	28.0%	
	+6	12877	25.6%	5616	52.5%	
SES						
	1	7778	15.4%	1261	11.8%	<0.001
	2	9221	18.3%	1520	14.2%	
	3	10236	20.3%	2451	22.9%	
	4	11047	21.9%	2398	22.4%	
	5	12088	24.0%	3074	28.7%	
Baseline exposure						
	<10	4516	9.0%	1050	9.8%	<0.002
	10-49	18622	37.0%	4064	38.0%	
	+50	57232	54.1%	5590	52.3%	

We found an increased risk of cerebrovascular related mortalities for the higher exposure groups. Participants exposed to 10-49 μg/L of well water were 1.20 (95% CI: 0.91 to 1.57) times more likely to die from cerebrovascular disease compared to participants who were exposed to <10 μg/L) and participants exposed to ≥50 μg/L of well water were 1.35 (95% CI: 1.03 to 1.75) times more likely to die from cerebrovascular disease compared to participants who were exposed to <10 μg/L (Table 3).

**Table 3:** Association of baseline exposure with mortality due to cerebrovascular disease for the total sample and by gender

**Baseline exposure (μg/L)**	**Deaths, No**	**Person-Years, No**	Rate*	**HR (95% CI)**		**P**_**trend**_
				**Unadjusted**	Adjusted**	
Male						
<10	36	15986	2.3	1.0	1.0	0.45
10-49	196	63824	3.1	1.37 (0.96- 1.95)	1.12 (0.78- 1.60)	
+50	271	17601	3.6	1.30 (0.92- 1.84)	1.07 (0.75- 1.51)	
Female						
<10	26	22212	1.2	1.0	1.0	0.00004
10-49	179	92538	1.9	1.65 (1.09- 2.49)	1.31 (0.87- 1.98)	
+50	325	24978	2.6	2.06 (1.38- 3.07)	1.72 (1.15- 2.57)	
Total						
<10	62	38198	1.6	1.0	1.0	0.00058
10-49	375	156362	2.4	1.48 (1.13- 1.93)	1.20 (0.92- 1.57)	
+50	596	42579	3.0	1.62 (1.24- 2.10)	1.35 (1.04- 1.75)	

*Crude death rate per 1000 person-years, **HR was adjusted with age, sex, education attainment and SES.

For men, multivariable adjusted HRs (95%) for well water arsenic concentrations <10, 10-49, and ≥50 μg/L were 1.0 (reference), 1.16 (0.78 to 1.59), and 1.06 (0.75 to 1.51) respectively and for women 1.0 (reference), 1.31 (0.87 to 1.98), and 1.72 (1.15 to 2.57), respectively.

**Figure 2:**
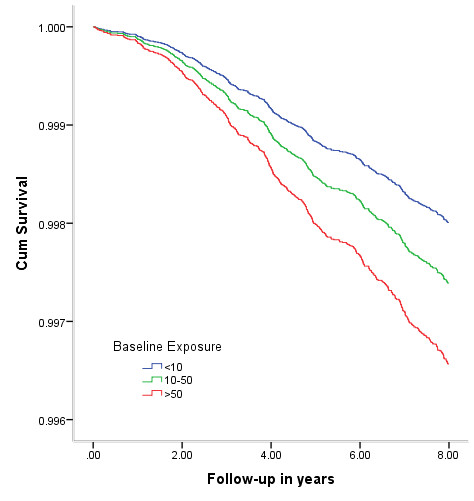
Cumulative survival of cerebrovascular mortality of women plotted against time for baseline arsenic exposure categories and adjusted for educational attainment, and SES.

This increased risk of strokemortality was much more pronounced in women than men. For women, the mortality rate for cerebrovascular disease was 117.1 per 100 000 person-years in people drinking water containing <10 μg/L compared with 238.3 per 100 000 person-years in people drinking with ≥10 μg/L. The test of trend was significant for women (P=0.00027) but not for men (p=0.99). Likewise, women exposed to ≥50 μg/L of well water were 1.69 (95% CI: 1.13 to 2.53) times more likely to die from stroke mortality compared to those who were exposed to <10 μg/L (Table 3) while risks were not statistically significantly increased in men. The overall survival curves by sex are shown in Figure 2. A significant test for trend was also observed for overall (P_trend_ =0.00058), for men (P_trend_=0.45) and for women (P_trend_=0.00004).

## Discussion

### Statement of principle findings

In our study, the risk of strokerelated morality increased with increasing arsenic exposure. To our knowledge, this is the first large population based longitudinal study with individual level arsenic exposure that focused on stroke mortality in Bangladesh or elsewhere. Our study highlights three main risks: i) We observed an increased risk of overall stroke mortality with increasing arsenic concentration in drinking water, with a significant trend test and risks in the range of 1.03-1.75 for the high arsenic groups ii) These risks were particularly marked in women rather than men and iii) These risks were also particularly marked for outcomes coded as sequelae of stroke, with increased risks, e.g. 1.64 (95% CI: 1.08-2.53) even at arsenic concentrations of 10-50 μg/L which are within the WHO permissible limit.

### Potential mechanism

The association between arsenic exposure and death from stroke is stronger among women than in men. Folate plays an important role of developing cerebrovascular disease [13]. Hyperhomocysteinemia, a proxy measure for B vitamins, has been related with low plasma folate and vitamin B12. Elevated hyperhomocysteinemia is an independent risk factor for stroke and vascular diseases [14]. The mechanism is not clear, but homocysteine causes atherogenesis and thrombogenesis via endothelial damage, focal vascular smooth muscle proliferation probably causing irregular vascular contraction, and coagulation abnormalities. Oxidative damage to the vascular endothelium and the proliferation of the vascular smooth muscle create a prothrombotic condition, which contributes to the development of premature atherosclerosis. Moreover, hyperhomocysteinemia has also been documented risk factor for cardiovascular disease and vascular dementia [15,16]. Contrary, many epidemiologic studies suggest that persons with impaired arsenic metabolism are at increased risk for diseases and this metabolism involves methylation to monomethylarsonic acid (MMA) and dimethylarsinic acid (DMA) by a folate-dependent process. Very recently folic acid supplementation causes improvement in arsenic metabolism [17,18]. Persons possessing polymorphisms in certain genes involved in folate metabolism excrete a lower proportion of urinary arsenic as DMA, which may influence susceptibility to arsenic toxicity, suggesting synergistic affect to increase the risk of stroke. Typically, Bangladeshi women are slender and short [19] as well as anemia and iron deficiencies are also prevalent [20,21].

The potential mechanism by which arsenic may increase vascular risk is still speculative. Both diabetes and hypertension have been associated with arsenic and are risk factors for stroke. The most informative studies regarding diabetes mellitus have been performed in Taiwan, Sweden, and Bangladesh in arsenic exposed population [22-25]. Reports indicate that arsenic induces renal insufficiency through cortical necrosis with haematuria, leukocyturia, and glucosuria [26] and that it inhibits the binding capability of the glucocorticoid receptor [27]. In addition, arsenic may damage the endothelial barrier in the vascular system and also activate leukocytes and platelets and thereby initiate plaque formation [28]. It is already documented that chronic arsenic exposure causes hypertension [29-34] and there are many biologically plausible routes for this. Firstly arsenic may directly accelerate atherosclerosis and increase production of reactive oxygen, [35] and nitrogen species [36]. Secondly inhibition of endothelial nitrogen oxide synthesis and enhanced vasoconstriction by arsenic.

Smoking is a well-known risk factor for stroke mortalities. However, we did not collect smoking information in this study. Razzaque et al found high prevalence of smoking (53·9% among male, 0·8% among female) in the adult population in Matlab [37]. In recent Matlab stroke study revealed no association with smoking (OR 1.41, 95% CI 0.82 to 2.45, P=0.22) [38].

### Strength and limitation

Strengths of our study include the large sample size, the collection of data on individual level baseline arsenic exposure, and the prospectively collected independent outcome data from the HDSS databases. These include larger outcome data over an extended follow-up time (over 1033 deaths recorded during more than 0·5 million person-years at risks). Secondly, standardized approaches to adjustment for several potential confounding factors, assessment of risk factors in 61,074 participants and information about cause-specific deaths from widely validated verbal autopsy methods contribute to the strength of the study. Selection and information biases were minimized by combining outcome data from the regular monthly surveillance at the household level with exposure data from the cross-sectional survey of arsenic concentrations in all tube-wells and interviews about drinking water history at the individual level. About 46% of the people (N=28,252) had exposure less than 50 μg/L which is larger in size than the total number of other cohort [HEALS (Health Effects Arsenic Longitudinal Study)] in Bangladesh [39].

Despite the strengths, several biases could persist owning to unmeasured or imprecisely measured potential confounding factors (smoking and betel squid history and blood pressure for example). The major weakness of the study is that exposure was assessed only once at baseline. Also, we considered only well water arsenic exposure and did not estimate exposure from other sources, such as diet. The unaccounted changes of As sources across the observational time is a weakness. Arsenic concentration in the tube-well water, as determined in 2002–2003, had remained similar during the follow-up period, we assume. Temporal variations of arsenic concentrations have not been extensively explored. Repeated water analyses were found to be fairly stable over a 3-year period in Bangladesh [40] and also in West Bengal [41]. Further, the British Geological Survey (BGS) analyzed random samples from all over the country for a 1.5-year period without showing any time trends [42]. In western Nevada in the United States, and northern Argentina the water arsenic concentrations remained about the same over periods of 10–20 years [43,44]. Moreover, in our study area, when following 61 randomly selected tube wells three times per year over a 3-year period, no systematic time trends could be shown [45]. Therefore, findings are to be interpreted under the assumption of constant source of As measured in 2003 and that the impact of possible changes is unknown. Considering smoking data, smoking is well known risk factor for stroke mortalities and very few women are reported to smoke in Bangladesh. Women use betal squid in rural areas and recently suggested is likely contributing to high blood pressure in Bangladeshi women [46]. Therefore, we separately analysed the data by sex. Thus we believe there is a true effect of arsenic exposure on death from stroke and do not believe our study findings would differ much with adjustment for smoking. To be a confounding factor for stroke people with high arsenic specifically smoke more which is unlikely.

## Conclusion

In an adult population, a dose-response relationship was observed between arsenic exposure and stroke mortality in this population, particularly in women. While arsenic mitigation programs are on-going, the resulting health effects of this catastrophe deserve urgent attention and resources. Despite limitation, because of uniqueness population-exposure data at individual level and the independent prospective demographic surveillance system covering 0·2 million population for about five decades, future research from this cohort will further strengthen our knowledge and help identify effective prevention program.

## Competing interest

All authors declare that they have no competing interests.

## Authors’ contributions

MR, NS analyzed the data and MR wrote the manuscript. NS, MY, KZ, AB, PKS contributed to the study design, data collection and critical review of draft manuscripts. MEC, SKH assisted with the statistical analysis, interpretation of data and critical review of draft manuscripts. All the authors read and approved the final manuscript.

## Pre-publication history

The pre-publication history for this paper can be accessed here:

http://www.biomedcentral.com/1471-2458/14/174/prepub
